# Comprehensive Analysis Reveals the Potential Regulatory Mechanism Between Ub–Proteasome System and Cell Cycle in Colorectal Cancer

**DOI:** 10.3389/fcell.2021.653528

**Published:** 2021-06-14

**Authors:** Zhiyuan Zhang, Jingwen Chen, Wentao Tang, Qingyang Feng, Jianmin Xu, Li Ren

**Affiliations:** Department of General Surgery, Zhongshan Hospital, Fudan University, Shanghai, China

**Keywords:** Ub–proteasome system, cell cycle, signature, colorectal cancer, bioinformatic

## Abstract

The ubiquitin (Ub)–proteasome system (UPS) is an important regulatory component in colorectal cancer (CRC), and the cell cycle is also characterized to play a significant role in CRC. In this present study, we firstly identified UPS-associated differentially expressed genes and all the differentially expressed protein-coding genes in CRC through three differential analyses. UPS-associated genes were also further analyzed via survival analysis. A weighted gene co-expression network analysis (WGCNA) was used to identify the cell cycle-associated genes. We used protein–protein interaction (PPI) network to comprehensively mine the potential mechanism of the UPS–cell cycle regulatory axis. Moreover, we constructed a signature based on UPS-associated genes to predict the overall survival of CRC patients. Our research provides a novel insight view of the UPS and cell cycle system in CRC.

## Introduction

The ubiquitin (Ub)–proteasome system (UPS) is identified to regulate the cellular protein by ubiquitination modification. Ub is an important component in the UPS. It consists of a 76-amino-acid protein and is usually highly conserved in eukaryotes (Zheng and Shabek, 2017). Among the UPS, three significant enzymes function as enzyme cascade to transmit the Ub to the substrate. The three vital enzymes are identified as the Ub-activating enzyme (E1), the Ub-conjugating enzyme (E2), and the Ub-protein ligase (E3) (Pickart, 2001). The UPS process can be summarized as follows: at first, E1 activates Ub and transmits it to E2 by an adenosine triphosphate-dependent way. Then, E3 mediates the last step by interacting with E2 that is carried with the Ub and recognizing a specific substrate. E3 are usually identified as the most crucial component among the three-enzyme cascade because the interaction between E3 and the substrate is highly specific, and the ubiquitination of the substrate mainly depends on the E3.

The UPS, especially E3, is also proved to contribute a lot in the process of cancers. Dysregulated E3 were widely reported to occur in diverse cancers. For example, HERC3 was reported to mediate the ubiquitination and the degradation of SMAD7 in glioblastoma (Li et al., 2019). RNF6 was indicated to induce the progression of colorectal cancer (CRC) through mediating ubiquitination of TLE3 (Liu et al., 2018). Moreover, E3 can regulate the downstream substrate including many oncogenes and tumor suppressors. P53 was reported to be degraded by the E3 ligase RING1 (Shen et al., 2018). Cancer is featured as an uncontrolled cell proliferation that is also regulated by many cell cycle-related regulators (Williams and Stoeber, 2012; Otto and Sicinski, 2017). These regulators can also be ubiquitination modified and degraded by E3. Thus, the UPS especially E3 may be the key regulators in the proliferation of cancer cells and may also become the therapeutic targets to address the uncontrolled proliferation of cells (Nakayama and Nakayama, 2006).

Colorectal cancer was ranked the top in the aspect of mortality and morbidity among diverse cancer types (Siegel et al., 2020). Given the crucial role of E3 in cancers, it is urgent and necessary to research the UPS in CRC. However, the integrated analysis of UPS or the interaction between UPS and cell cycle-related regulators in CRC is still blank. In this present study, we integrated and analyzed the UPS, especially the E3 ligase in CRC; moreover, we constructed a prognostic signature based on UPS-associated regulators and further depicted the potential interactions between UPS and specific cell cycle-related genes in CRC. We provide a novel insight into the UPS and the latent interaction between cell cycle-associated genes and the UPS in the field of CRC.

## Materials and Methods

### Acquisition and Processing of the Raw Data

The raw microarray data and relevant clinical information of CRC patients that were based on TCGA (The Cancer Genome Atlas) database and the raw microarray data of normal colon samples that were based on the GTEx (Genotype-Tissue Expression) database were downloaded from XENA^1^. Then, the data from TCGA and data from GTEx were normalized and combined based on the description of the website. The UPS-associated genes were obtained from an article published by Ge et al. (2018). The detailed information of these UPS-associated genes are provided in Supplementary Table 1.

### Identification of Differentially Expressed Genes

An analysis differentially expressed genes was conducted three times according to the diverse grouping of samples. The three comparative groups were set as follows: GTEx normal colon samples VS. TCGA CRC-adjacent normal colon samples; TCGA CRC-adjacent normal colon samples VS. TCGA CRC samples; and GTEx normal colon samples combined with TCGA CRC-adjacent normal colon samples VS. TCGA CRC samples. Wilcoxon test was utilized to perform the differential analysis. The selection criterion was set as FDR < 0.05.

### Survival Analysis

Survival analysis was performed on the UPS-associated genes based on the expression pattern and clinical information [overall survival (OS) information] from TCGA. The Kaplan–Meier plot was used to visualize the results and the median expression of the relevant gene was set as the cutoff. The log-rank test was used to analyze the results, and a *P*-value < 0.05 was identified as statistically significant.

### Gene Set Enrichment Analysis and Single-Sample Gene Set Enrichment Analysis

A gene set enrichment analysis (GSEA) was used to identify the biological pathways that might play significant roles in the process of CRC. The input reference gene sets were all the protein-coding genes from the intersection of the three differential analyses. A single-sample gene set enrichment analysis (ssGSEA) was conducted to calculate the score for individual samples based on a specific reference.

### Weighted Gene Co-Expression Network Analysis

A weighted gene co-expression network analysis (WGCNA) was performed through the R package WGCNA in R based on the relevant instructions (Langfelder and Horvath, 2008). Parameters used in the WGCNA process were set as default. Several cell cycle-related pathways that were statistically significant in GSEA were set as references to calculate the correlation between genes and those pathways. A *P*-value < 0.05 and a correlation value >0.7 were identified as statistically significant. Before WGCNA, we screened out the differentially expressed and OS-related genes. Firstly, 3 times differential analysis same as previously performed was carried out based on the whole genes, the criterion was set as FDR < 0.05. Then the results were subjected to COX analysis. The criterion was set as *P* < 0.05 and HR < 1.2 or HR < 0.05.

### Construction of Protein–Protein Interaction Network

The protein–protein interaction (PPI) network was drawn based on the STRING^2^. The input genes were differentially expressed UPS-associated genes obtained from the three differential analyses and the cell cycle-related genes that were obtained from the WGCNA. The PPI network was divided into four parts, each module representing E2 and E3 (E3 adaptor and E3 activity, respectively) and the potential cell cycle-associated substrates. The PPI network was visualized by Cytoscape (3.8.2).

### Construction and Internal Validation of a Prognostic Signature Based on Differentially Expressed UPS-Associated Genes

The TCGA patients were randomly divided into two parts at a ratio of 7:3. The 70% of the patients were identified as the internal training set and the 30% of the patients were identified as the internal validation set. A least absolute shrinkage and selection operator (LASSO) analysis was conducted to screen out the variates for further analysis. A multivariate Cox regression analysis was performed to construct the signature. The accuracy of the signature was validated in the internal validation set through the receiver operating characteristic (ROC) curve, risk score analysis, and Kaplan–Meier survival analysis. A *P*-value < 0.05 was identified as statistically significant.

## Results

### Differentially Expressed UPS-Associated Genes in CRC

**FIGURE 1:**
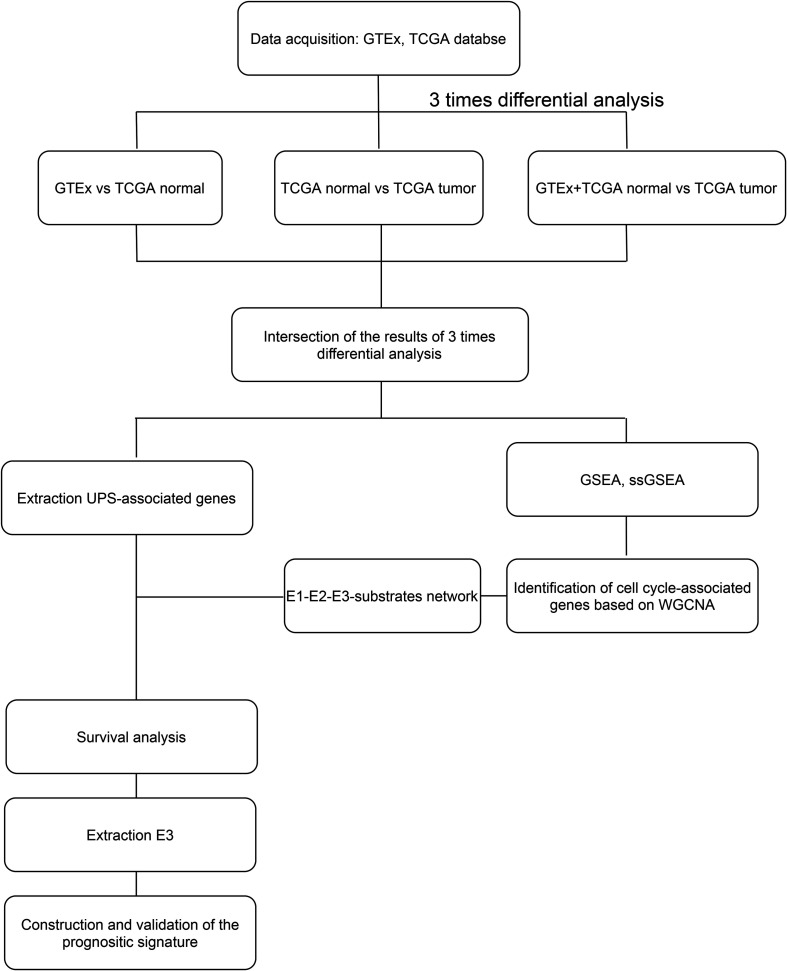
Flow chart of the entire work.

**FIGURE 2:**
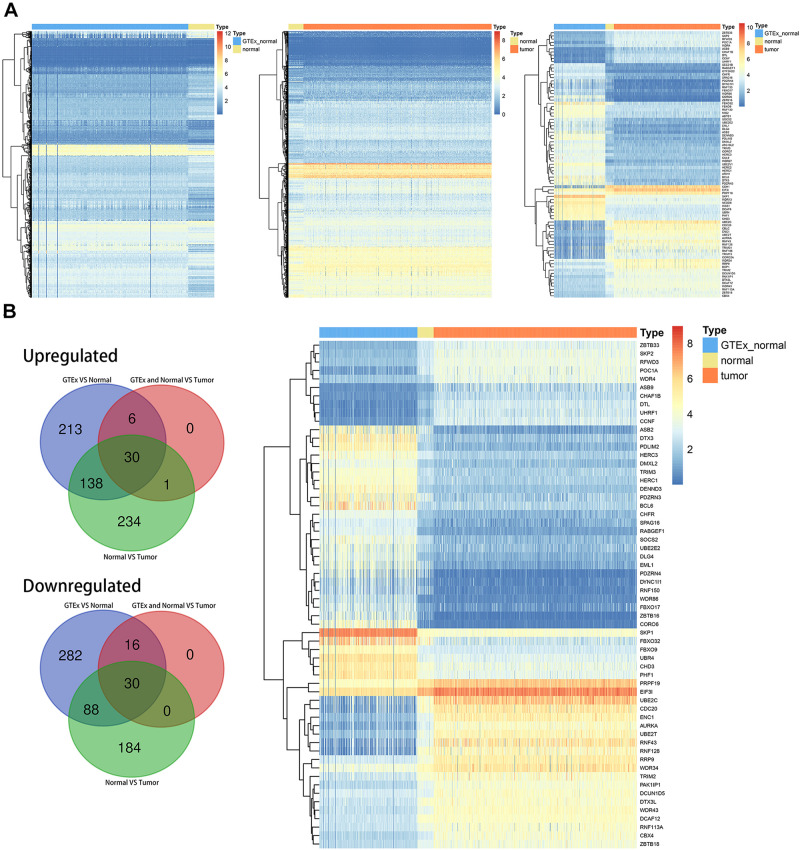
Differentially expressed ubiquitin (Ub)–proteasome system (UPS)-associated genes in colorectal cancer (CRC). **(A)** Heatmaps depicting the expression landscape of differentially expressed UPS-associated genes in three comparative groups. **(B)** Numbers of intersected differentially expressed UPS-associated genes in three comparative groups (left panel) and the expression pattern of 60 differentially expressed UPS-associated genes in three subgroups of datasets (right panel).

**FIGURE 3:**
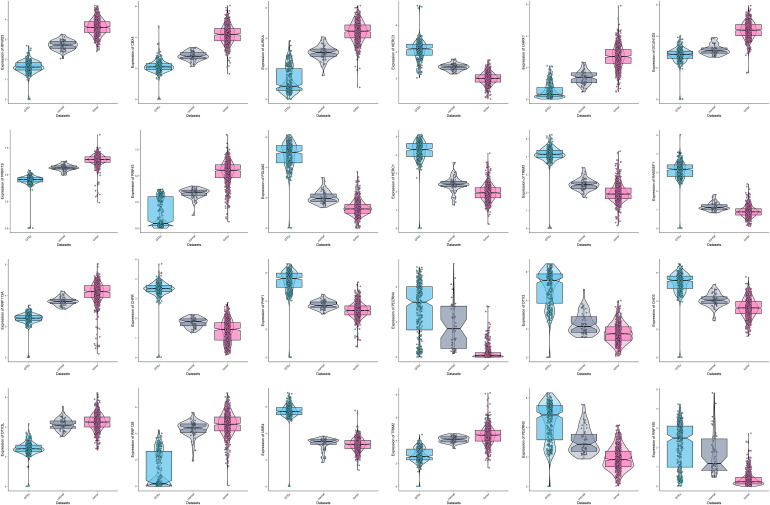
The expression pattern of 24 differentially expressed E3 genes in three subgroups of datasets.

The entire flow of the research is presented in Figure 1. We first identified differentially expressed UPS-associated genes through three differential analyses according to the diverse grouping of the entire patients. We first compared the normal colon samples from GTEx with CRC-adjacent normal samples in TCGA. We then compared the CRC-adjacent normal samples with CRC samples in TCGA. Finally, we combined normal colon samples from GTEx with CRC-adjacent normal samples from TCGA and identified the differentially expressed genes between those samples and CRC samples in TCGA. A heatmap is depicted in Figure 2A. Afterward, we intersected the upregulated genes and the downregulated genes from the three differential analyses, respectively. We got 30 upregulated and 30 downregulated UPS-associated genes, respectively (Figure 2B). The expression landscape of these 60 dysregulated UPS-associated genes in three diverse groups is shown in Figure 2B. The results of the differential analysis when comparing normal colon samples in GTEx plus CRC-adjacent normal samples with CRC samples in TCGA are demonstrated in Table 1. Among these 60 UPS-associated genes, 24 genes were identified as E3. We also used violin plots to depict the expression pattern of 24 E3 in Figure 3. The detailed information of the three differential analyses of the 24 E3 is presented in Supplementary Table 2.

**TABLE 1:** The results of the differential analysis when comparing normal colon samples in GTEx plus CRC-adjacent normal samples with CRC samples in TCGA.

**Gene**	**Category**	**LogFC**	***P*-value**	**FDR**	**Gene**	**Category**	**LogFC**	***P*-value**	**FDR**
DYNC1I1	E3 adaptor	–1.70	3.18E-99	1.23E-98	DTL	E3 adaptor	2.10	1.51E-144	1.20E-142
BCL6	E3 adaptor	–2.33	1.27E-102	5.49E-102	WDR43	E3 adaptor	1.97	2.88E-148	6.27E-146
FBXO17	E3 adaptor	–1.83	1.43E-107	7.14E-107	ENC1	E3 adaptor	3.31	2.15E-151	1.87E-148
FBXO32	E3 adaptor	–2.68	4.24E-112	2.55E-111	RNF150	E3 activity	–1.72	4.11E-106	2.00E-105
EML1	E3 adaptor	–2.14	3.75E-112	2.27E-111	PDZRN3	E3 activity	–2.29	3.46E-109	1.84E-108
DLG4	E3 adaptor	–1.83	1.24E-114	8.21E-114	TRIM2	E3 activity	1.55	2.24E-110	1.26E-109
WDR86	E3 adaptor	–1.80	7.74E-115	5.23E-114	UBR4	E3 activity	–2.13	4.27E-113	2.64E-112
FBXO9	E3 adaptor	–1.71	7.21E-119	5.61E-118	RNF128	E3 activity	3.07	7.13E-116	4.89E-115
DCAF12	E3 adaptor	1.62	1.07E-119	8.55E-119	DTX3L	E3 activity	1.53	4.04E-116	2.82E-115
SPAG16	E3 adaptor	–1.61	5.64E-122	4.77E-121	CHD3	E3 activity	–1.58	2.23E-116	1.58E-115
ASB9	E3 adaptor	1.65	2.85E-122	2.44E-121	DTX3	E3 activity	–2.84	4.37E-121	3.59E-120
DMXL2	E3 adaptor	–1.79	6.72E-123	5.97E-122	PDZRN4	E3 activity	–2.40	1.60E-121	1.33E-120
SKP1	E3 adaptor	–2.85	4.00E-123	3.71E-122	PHF1	E3 activity	–1.84	8.76E-123	7.70E-122
CORO6	E3 adaptor	–3.28	8.24E-125	8.45E-124	CHFR	E3 activity	–1.88	1.61E-124	1.61E-123
ASB2	E3 adaptor	–2.97	3.52E-125	3.83E-124	RNF113A	E3 activity	1.52	4.41E-127	5.42E-126
WDR34	E3 adaptor	1.59	1.04E-125	1.17E-124	RABGEF1	E3 activity	–1.92	7.08E-128	8.94E-127
DENND3	E3 adaptor	–2.53	2.62E-127	3.26E-126	TRIM3	E3 activity	–1.89	5.51E-128	7.05E-127
POC1A	E3 adaptor	2.27	1.99E-129	2.84E-128	HERC1	E3 activity	–1.99	5.24E-128	6.81E-127
CDC20	E3 adaptor	3.56	1.29E-135	2.61E-134	PDLIM2	E3 activity	–3.11	1.10E-135	2.29E-134
ZBTB18	E3 adaptor	1.59	2.64E-136	5.75E-135	RNF43	E3 activity	3.66	1.07E-136	2.44E-135
PAK1IP1	E3 adaptor	1.76	3.81E-137	9.48E-136	PRPF19	E3 activity	1.73	4.73E-138	1.47E-136
EIF3I	E3 adaptor	1.68	2.69E-137	7.24E-136	DCUN1D5	E3 activity	1.51	2.59E-143	1.50E-141
SOCS2	E3 adaptor	–2.06	2.69E-137	7.24E-136	UHRF1	E3 activity	2.09	7.16E-144	4.80E-142
RRP9	E3 adaptor	2.25	8.25E-139	2.87E-137	HERC3	E3 activity	–1.95	9.94E-145	8.66E-143
WDR4	E3 adaptor	1.68	1.03E-139	3.90E-138	AURKA	E3 activity	2.92	2.37E-146	2.58E-144
ZBTB33	E3 adaptor	1.77	2.90E-140	1.20E-138	CBX4	E3 activity	1.98	1.54E-146	2.23E-144
CHAF1B	E3 adaptor	1.59	1.06E-140	4.60E-139	RFWD3	E3 activity	1.83	1.45E-149	6.32E-147
ZBTB16	E3 adaptor	–2.55	6.16E-141	3.16E-139	UBE2E2	E2	–1.60	1.99E-89	6.14E-89
SKP2	E3 adaptor	1.89	3.44E-143	1.87E-141	UBE2T	E2	2.87	5.37E-144	3.90E-142
CCNF	E3 adaptor	2.08	9.83E-144	6.12E-142	UBE2C	E2	4.27	1.28E-148	3.73E-146

### Identification of 12 OS-Related Genes From 60 Dysregulated UPS-Associated Genes

**FIGURE 4:**
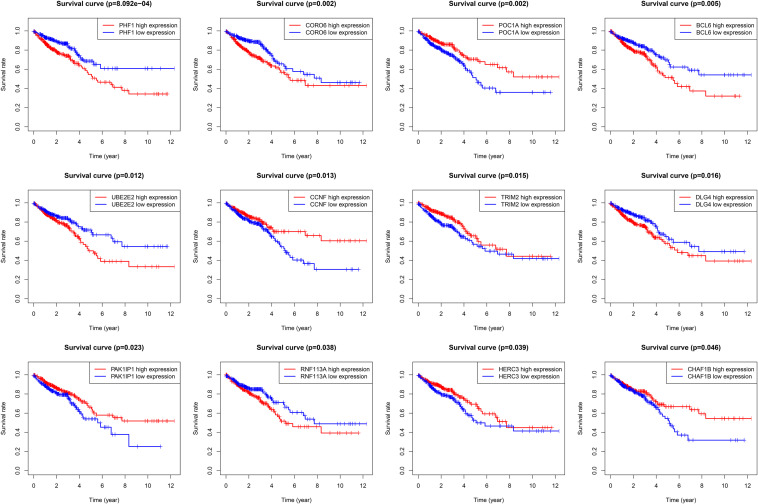
Kaplan–Meier plots of 12 overall survival-related differentially expressed UPS-associated genes.

Based on the clinical information (OS) in TCGA, we conducted a survival analysis through Kaplan–Meier plots, and the log-rank test was utilized as the analytical method. We identified 12 genes from the 60 dysregulated UPS-associated genes as OS-related genes (statistically significant) (Figure 4). They were BCL6, CCNF, CHAF1B, CORO6, DLG4, HERC3, PAK1IP1, PHF1, POC1A, RNF113A, TRIM2, and UBE2E2.

### Identification of Potential Significant Biological Pathways in the Process of CRC

**FIGURE 5:**
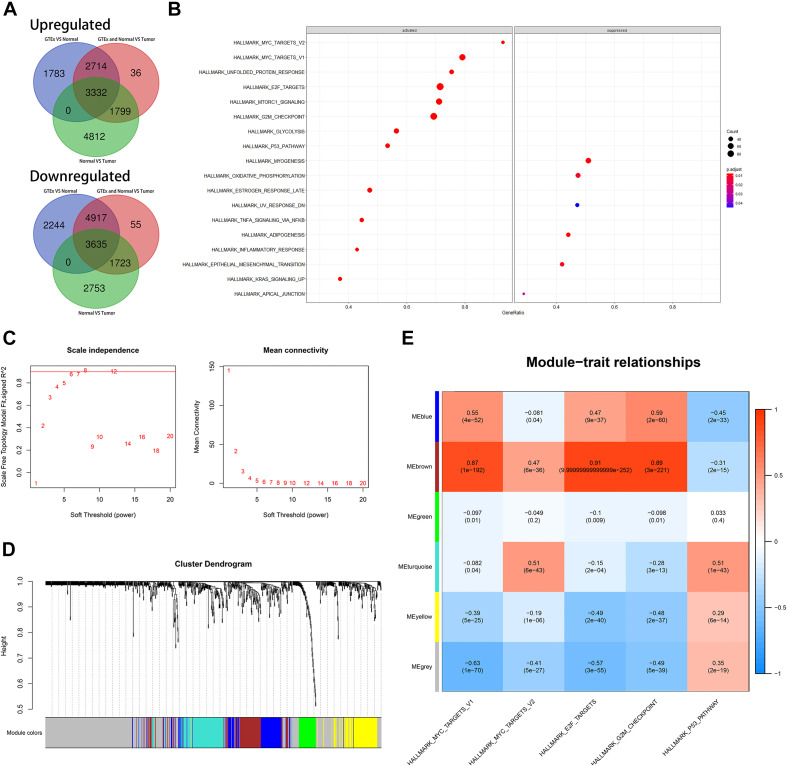
Identification of latent biological pathways that might mainly be involved in the process of CRC and identification of cell cycle-associated genes. **(A)** The number of intersected differentially expressed genes among diverse comparative groups. **(B)** A gene set enrichment analysis (GSEA) of intersected differentially expressed genes among diverse comparative groups. **(C)** Confirmation of suitable soft-thresholding power to construct a scale-free network. **(D)** Identification of co-expression models and assignments of the corresponding color. **(E)** Correlation analysis between modules and relevant parameters.

We identified dysregulated protein-coding genes through three differential analyses. The comparative groups were also set as described in the “Materials and Methods” section. We also intersected the upregulated genes and downregulated genes, respectively. Finally, we obtained 3,332 upregulated genes and 3,635 downregulated genes (Figure 5A). GSEA was performed based on these genes. The result of GSEA indicated that cell cycle might be the significant process during the progression of CRC because MYC_TARGETS, E2F_TARGETS, G2M_CHECKPOINT, and P53_PATHWAY were important regulator during the process of cell cycle (Figure 5B). ssGSEA was also conducted, and the cell cycle-related biological pathways obtained from the GSEA were set as reference.

### Identification of Potential Cell Cycle-Associated Substrates

Before WGCNA, we screened out the differentially expressed and OS-related genes as described before. We performed WGCNA as mentioned in *MATERIALS AND METHODS*. As depicted in Figure 5C, the suitable soft threshold (power) was set as eight. Through the WGCNA process, we identified six modules, and the cell cycle-related biological pathways obtained from the GSEA based on the all dysregulated protein-coding genes were set as reference. Then, MEbrown was identified as the most correlated module with cell cycle-related pathways. There are a total of 97 genes in MEbrown and they were identified as potential cell cycle-associated substrates (Figures 5D,E).

### Identification of the PPI Network

**FIGURE 6:**
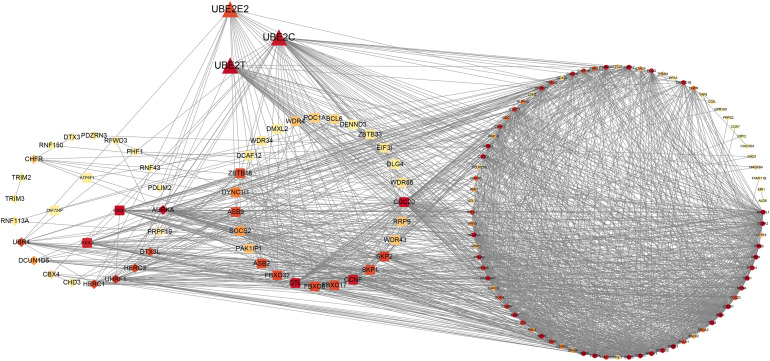
Protein–protein interaction network of UPS and potential cell cycle-associated substrates.

Based on the dysregulated 60 UPS-associated and the potential cell cycle-associated substrates obtained from WGCNA, we conducted the construction of the PPI network. The entire network is shown in Figure 6, and the details of the network are demonstrated in Supplementary Table 3. We divided the entire network into four modules as dysregulated E2, dysregulated E3 adaptor, dysregulated E3 activity, and potential cell cycle-associated substrates. Detailed information of genes involved in the PPI is shown in Supplementary Table 4.

### Construction of the Prognostic Signature Based on 60 Dysregulated UPS-Associated Genes

**FIGURE 7:**
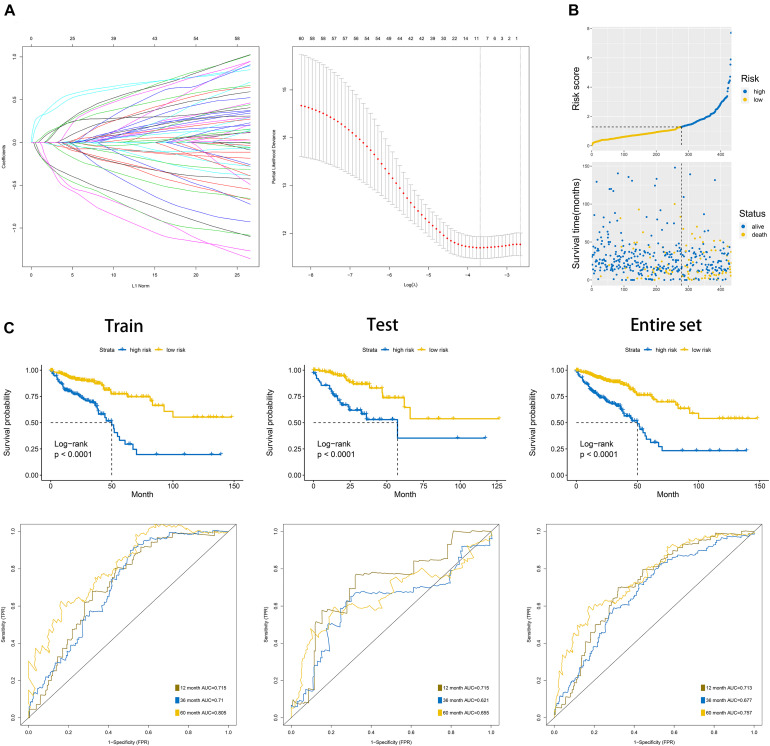
Construction of the overall survival prognostic signature based on 60 differentially expressed UPS-associated genes in CRC. **(A)** Results of the least absolute shrinkage and selection operator (LASSO) analysis. **(B)** Risk score analysis of the signature in the internal training dataset. **(C)** Kaplan–Meier plots and area under the curve (AUC) of receiver operating characteristic (ROC) curve in the internal training, internal test, and entire dataset.

We randomly divided the entire TCGA CRC patients into two parts at a ratio of 7:3; moreover, LASSO and multivariate cox regression analyses were performed to construct the signature based on the 70% of patients. The results of the univariate Cox analysis based on 60 differentially expressed UPS-associated genes are presented in Table 2. The efficiency was evaluated through a risk score analysis, Kaplan–Meier survival analysis, and ROC. The efficiency was also validated in the 30% of the patients through equivalent methods (Figures 7A–C). The formula was risk score = exp of CCNF × (−0.545) + exp of ZBTB18 × (−0.502) + exp of RNF113A × 0.687 + exp of CHD3 × 0.347 + exp of PHF1 × 0.401 + exp of BCL6 × 0.368 + exp of ZBTB16 × (−1.216) + exp of SKP1 × (−0.588). Then, the cutoff between high risk and low risk was 1.282. The areas under the curve (AUC) of ROC at 1, 3, and 5 years in the internal training set were 0.715, 0.71, and 0.805, respectively. The AUC of ROC at 1, 3, and 5 years in the internal test set were 0.715, 0.621, and 0.655, respectively. In the entire TCGA dataset, the AUC of ROC at 1, 3, and 5 years were 0.713, 0.677, and 0.757, respectively.

**TABLE 2:** The results of univariate Cox analysis based on 60 differentially expressed UPS-associated genes.

**Gene**	**HR**	**OR (95% CI high)**	**OR (95% CI low)**	***P*-value**	**Gene**	**HR**	**OR (95% CI high)**	**OR (95% CI low)**	***P*-value**
PHF1	1.81	1.32	2.47	0.00	SKP2	0.84	0.62	1.14	0.27
DLG4	1.64	1.17	2.30	0.00	ASB9	0.91	0.75	1.09	0.30
CCNF	0.65	0.48	0.88	0.01	DTL	0.85	0.62	1.17	0.32
BCL6	1.41	1.10	1.82	0.01	DENND3	1.24	0.79	1.93	0.34
WDR86	1.93	1.15	3.25	0.01	FBXO17	1.12	0.88	1.42	0.37
TRIM2	0.74	0.58	0.94	0.01	UBE2T	0.89	0.69	1.16	0.40
CHD3	1.42	1.07	1.88	0.01	PDZRN3	1.09	0.87	1.36	0.47
CORO6	2.54	1.17	5.49	0.02	DTX3L	0.89	0.63	1.26	0.52
ZBTB18	0.72	0.54	0.95	0.02	FBXO9	0.84	0.50	1.41	0.52
UBE2E2	1.29	1.02	1.64	0.03	ASB2	1.08	0.83	1.41	0.55
RNF43	0.85	0.73	0.99	0.03	PDLIM2	1.12	0.78	1.60	0.55
DCAF12	0.58	0.35	0.96	0.03	EML1	1.11	0.79	1.55	0.55
DTX3	1.29	1.01	1.65	0.04	UBE2C	0.94	0.76	1.17	0.59
CHAF1B	0.72	0.52	1.00	0.05	RNF128	0.95	0.78	1.16	0.61
POC1A	0.78	0.61	1.00	0.05	ENC1	0.94	0.71	1.24	0.65
RNF113A	1.36	0.99	1.86	0.06	SPAG16	0.94	0.71	1.24	0.67
AURKA	0.79	0.60	1.04	0.09	RRP9	0.95	0.72	1.25	0.71
WDR43	0.73	0.50	1.08	0.11	CHFR	1.06	0.77	1.45	0.73
SKP1	0.73	0.49	1.09	0.13	CBX4	1.05	0.79	1.40	0.73
DCUN1D5	0.76	0.54	1.08	0.13	UBR4	0.93	0.63	1.39	0.73
PAK1IP1	0.78	0.55	1.09	0.14	SOCS2	0.95	0.68	1.34	0.78
RFWD3	0.72	0.46	1.13	0.16	HERC1	0.94	0.61	1.45	0.79
PDZRN4	1.28	0.89	1.83	0.18	WDR34	1.03	0.82	1.30	0.79
DYNC1I1	1.26	0.89	1.78	0.19	PRPF19	1.04	0.71	1.54	0.84
CDC20	0.87	0.71	1.07	0.19	FBXO32	1.02	0.83	1.25	0.85
HERC3	0.76	0.50	1.15	0.19	TRIM3	1.03	0.72	1.49	0.85
WDR4	0.82	0.61	1.11	0.20	DMXL2	1.03	0.72	1.47	0.88
RNF150	1.28	0.83	1.98	0.26	RABGEF1	1.04	0.57	1.88	0.90
EIF3I	0.84	0.62	1.14	0.26	ZBTB33	0.99	0.72	1.36	0.95
UHRF1	0.86	0.66	1.12	0.27	ZBTB16	1.00	0.54	1.84	0.99

## Discussion

Colorectal cancer is identified as one digestive system cancer type that has high morbidity and mortality (Siegel et al., 2020). Although the treatment of CRC progresses a lot during recent years, the potential regulatory mechanism of CRC is not clearly elucidated yet. The cell cycle is a crucial component in biological development. The cell cycle can be summarized into four phases: in the S phase, DNA replication is frequent; in the M phase, a single cell can divide into two daughter cells. There are also two gap phases between S and M, which can be characterized as G1 and G2. It is widely recognized that G1 is a phase in which a cell can be sensitive to stimulation of growth. G2 is the phase after S and specific for cell entering mitosis. Moreover, cancer is characterized as uncontrolled cell proliferation (Kastan and Bartek, 2004; Massagué, 2004). Uncontrolled cancer cell proliferation is also commonly observed in CRC. Zhang Z. et al. (2018) found that miR-1258 could regulate the CRC cell proliferation via regulating the cell cycle. Moreover, there was also a prognostic signature based on cell cycle specific in colon cancer (Zhang et al., 2020a). These studies implied that the cell cycle also has a significant function in the process of CRC. However, the detailed mechanism of the regulation of the cell cycle in CRC is not clear until now, and according to the vital role of the cell cycle in CRC, it is urgent to demonstrate the potential regulatory mechanism of the cell cycle in CRC.

The Ub–UPS is proved to be involved in the regulation of the process of many cancers. It can be summarized into many regulatory mechanisms. Firstly, the proteins that are involved in the UPS can be oncogenes or tumor suppressors. For instance, HERC3 was reported to be a tumor suppressor via regulating SMAD7 in glioblastoma (Li et al., 2019). RNF6 was reported to induce the progression of CRC through mediating ubiquitination of TLE3 (Liu et al., 2018). Secondly, many oncogenes or tumor suppressors can be regulated by E3, and P53 was indicated to be degraded by the E3 ligase RING1 (Shen et al., 2018). Given the important role of UPS in cancer, it is also urgent to comprehensively analyze the regulatory mechanism of UPS in CRC.

Given the important role of cell cycle and UPS in CRC, we comprehensively analyzed differentially expressed UPS-associated genes in CRC through three differential analyses. Moreover, we discovered that the cell cycle is one of the most important biological processes in the progression of CRC. We also used WGCNA to identify some cell cycle-associated genes that are specific in CRC. Furthermore, we used the differentially expressed UPS-associated genes and cell cycle-associated genes to construct the PPI network. Afterward, we used UPS-associated genes to construct the OS prognostic signature in CRC with relative considerable AUC.

Among the UPS, E3 ligase is undoubtedly the most important component because the interaction between E3 and the substrate is highly specific, and the ubiquitination of the substrate mainly depends on the E3. In this research, we found a total of 60 differentially expressed UPS-associated genes, among them, 24 genes were identified as E3 ligases. Among these 24 differentially expressed E3 ligases, many were proved to have relevant regulatory roles in the process of CRC. For example, AURKA was upregulated by ARID3 in CRC (Tang et al., 2020). CBX4 was reported to involve in the process of long non-coding RNA RAMS11 regulating the metastasis of CRC (Silva-Fisher et al., 2020). ASB8 was reported to be controlled by miR-452 in CRC cells (Mo and Chae, 2021). FBXO45 could be regulated by RP11 through the Siah1-Fbxo45/Zeb1 axis (Wu et al., 2019). TRIM27 was also reported to be an oncogene in CRC (Zhang Y. et al., 2018). Previous researches also confirmed that our analysis was reliable. Combing the results of survival analysis and the results of differential analysis, we found that HERC3 is an E3 ligase that owns the same trend of survival analysis and differential analysis, indicating the potential research value of HERC3 in CRC.

Through three differential analyses, we identified 3,323 upregulated genes and 3,635 downregulated genes in CRC. GSEA indicated that the cell cycle is an important component in CRC. Via WGCNA, we also identified cell cycle-associated genes specific in CRC. Moreover, the PPI network based on UPS-associated genes and cell cycle-associated genes provided many latent research orientations for the mechanism of uncontrolled cell proliferation in CRC.

Finally, we constructed an OS-associated signature based on the 60 differentially expressed UPS-associated genes. However, the signature still lacks enough validation, and this limitation is also a novel research direction for us. Compared with other signatures in CRC, our signature can predict the prognosis in colon cancer and rectal cancer with a considerate AUC; other signatures mainly focus on one cancer type only (colon cancer or rectal cancer) (Zhang et al., 2020a,b,c,d, 2021). Among these genes involved in the signature, some of them were previously reported to perform latent jobs in the progression of cancer. ZBTB18 was reported to be upregulated by circTP63 and further promote hepatocellular carcinoma progression (Wang and Che, 2021). RNF113A was revealed to promote the proliferation, migration, and invasion in esophageal squamous cell carcinoma (Wang et al., 2018). SKP1 was demonstrated to be involved in an axis to promote bladder cancer proliferation and is controlled by circGLIS3 (Wu et al., 2021).

In conclusion, we used bioinformatic analysis to reveal the potential regulatory mechanism between UPS-associated genes and potential cell cycle-related substrates specific in CRC. Besides, we constructed a prognostic signature based on the UPS-associated genes. Our research provides a novel insight of the UPS and cell cycle system in CRC.

## Data Availability Statement

The raw microarray data and relevant clinical information of CRC patients that were based on TCGA (The Cancer Genome Atlas) database, and raw microarray data of normal colon samples that were based on GTEx (The Genotype-Tissue Expression) database were downloaded from XENA (http://xena.ucsc.edu/).

## Ethics Statement

The studies involving human participants were reviewed and approved by the data was obtained from TCGA and GTEx database. The patients/participants provided their written informed consent to participate in this study. Written informed consent was obtained from the individual(s) for the publication of any potentially identifiable images or data included in this article.

## Author Contributions

ZZ, JX, and LR designed and conducted the study. ZZ and QF wrote the manuscript. JC and WT helped to improve and design the study. All authors contributed to the article and approved the submitted version.

## Conflict of Interest

The authors declare that the research was conducted in the absence of any commercial or financial relationships that could be construed as a potential conflict of interest.

## Footnotes

1
http://xena.ucsc.edu/

2 https://string-db.org/

## Supplementary Material

Supplementary Table 1 Detailed information of UPS-associated genes.

Supplementary Table 2 Detailed information of three differential analyses of the 24 E3.

Supplementary Table 3 Detailed interactions in the PPI network.

Supplementary Table 4 Detailed information of genes involved in the PPI.
